# Reversal of Platelet Inhibition in Patients Receiving Ticagrelor

**DOI:** 10.31083/j.rcm2309300

**Published:** 2022-09-05

**Authors:** Piotr Adamski, Grzegorz Skonieczny, Tomasz Hajdukiewicz, Adam Kern, Jacek Kubica

**Affiliations:** ^1^Department of Cardiology and Internal Medicine, Collegium Medicum, Nicolaus Copernicus University, 85-094 Bydgoszcz, Poland; ^2^Clinic of Cardiology and Cardiac Care Unit Department, Provincial Polyclinic Hospital, 87-100 Toruń, Poland; ^3^Department of Cardiology and Department of Cardiological Intensive Care, Provincial Hospital, 82-300 Elbląg, Poland; ^4^Department of Cardiology and Internal Medicine, University of Warmia and Mazury, 10-082 Olsztyn, Poland; ^5^Department of Cardiology, Regional Specialist Hospital, 10-082 Olsztyn, Poland

**Keywords:** antidote, bentracimab, MEDI2452, PB2452, platelet transfusion, ticagrelor

## Abstract

Antiplatelet treatment is one of the pillars of contemporary therapy in acute coronary syndromes. It is based on dual antiplatelet therapy (DAPT) consisting of aspirin and a P2Y12 receptor inhibitor. Antiaggregatory treatment reduces ischemic events, but at cost of increased bleeding rates. As a result of irreversible inhibition of platelet P2Y12 receptors, the antiplatelet action of clopidogrel and prasugrel is prolonged for the lifespan of thrombocytes and lasts up to 7 days. The antiaggregatory effect of ticagrelor may persist up to 5 days despite its reversible nature of P2Y12 receptor inhibition. These pharmacodynamic properties may prove problematic in patients requiring immediate reversal of antiplatelet effects due to severe or life-threatening bleeding, or in presence of indications for an urgent surgery. The current review summarizes available knowledge on different strategies of restoring platelet function in patients treated with ticagrelor. Non-specific methods are discussed, including platelet transfusion, human albumin supplementation and hemadsorption. Finally, bentracimab, the first specific antidote for ticagrelor, and in fact against any antiplatelet agent, is described.

## 1. Introduction

Antiplatelet treatment is one of the pillars of contemporary therapy in acute coronary syndromes (ACS). It substantially improves the clinical outcomes in ACS, mainly due to reduction of ischemic events. Notwithstanding, antiplatelet treatment increases the incidence of bleeding, which can range from nonsignificant and not requiring medical contact to fatal hemorrhages [1].

Antiaggregatory therapy in ACS is based on dual antiplatelet therapy (DAPT) consisting of aspirin and a P2Y12 receptor inhibitor [2]. Standard duration of DAPT after ACS is 12 months, unless contraindications or excessive bleeding risk exist. Duration (shortening or extension) and type of antiplatelet treatment following ACS should consider individual ischemic and bleeding risks [1]. In general ACS population potent P2Y12 receptor inhibitors, ticagrelor or prasugrel, are recommended over clopidogrel [2, 3, 4]. Both of these agents provide stronger and more predictable antiaggregatory effect, and in result improved clinical outcomes, compared with clopidogrel [2]. However, in the landmark trials both ticagrelor and prasugrel were related with greater incidence of bleeding compared with the latter [5, 6]. Additionally, non-adherence to DAPT after ACS, and especially early discontinuation of antiplatelet treatment, is associated with significantly increased risk of major adverse cardiovascular events [7].

The antiplatelet effect of oral P2Y12 receptor antagonists extends to at least several days after intake of the last dose. Ticagrelor provides stronger platelet inhibition than clopidogrel even in reduced doses [8]. Due to irreversible inhibition of platelet P2Y12 receptors, the antiaggregatory action of clopidogrel and prasugrel is prolonged for the lifespan of thrombocytes and lasts up to 7 days [1, 9]. The antiplatelet effect of ticagrelor may persist up to 5 days, despite its reversible nature of P2Y12 inhibition [10]. These pharmacodynamic properties may prove problematic in patients requiring immediate reversal of antiplatelet effects due to severe or life-threatening bleeding, or in presence of indications for an urgent surgery. Detailed data are not available, but it is estimated that up to 25% of patients undergoing coronary stenting may require non-cardiac surgery within 5 years after percutaneous coronary intervention (PCI) [11]. Thus, feasibility of prompt restoration of platelet function is of great importance in patients receiving antiplatelet agents.

Currently there are no commercially available specific antidotes for any of oral P2Y12 receptor antagonists. Due to irreversible nature of adenosine diphosphate (ADP) receptor binding, antidotes for thienopyridines (clopidogrel, prasugrel) are not very likely to be developed in the near future. Conversely, because of reversible P2Y12 inhibition yielded by ticagrelor, recovery of platelet function in patients receiving this antiplatelet agent should be more feasible.

The aim of this review was to summarize available data on non-specific and specific methods of platelet function recovery in patients receiving ticagrelor.

## 2. Ticagrelor

**Fig. 1.**
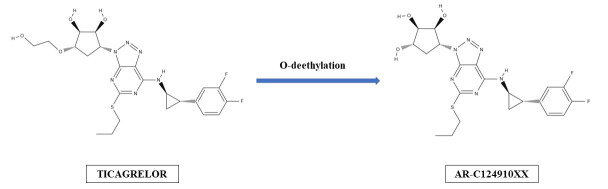
**Hepatic formation of ticagrelor’s active metabolite (AR-C124910XX)**.

Ticagrelor is a P2Y12 receptor inhibitor that belongs to cyclopentyl-triazolo-pyrimidine group and binds to platelet P2Y12 receptors in a reversible and noncompetitive manner. It is an active drug, but it also undergoes hepatic metabolism and is transformed into 10 metabolites, of which one exerts antiplatelet potency equal to one of the parent drug (Fig. 1). Ticagrelor is expeditiously absorbed after oral intake and is characterized by rapid onset of antiaggregatory effect. In stable setting time to maximal concentration usually does not exceed 2 hours [10]. However, in patients with ACS both absorption and antiplatelet action of ticagrelor can be reduced and delayed for few hours, especially if morphine is administered [12, 13, 14]. Elimination half-time of ticagrelor is 7.7–13.1 hours and duration of platelet inhibition lasts for 3–5 days [10]. One of the most important differences between thienopyridines and ticagrelor is the reversibility of P2Y12 receptor inhibition by ticagrelor. Subsequently, recommended time of P2Y12 receptor antagonist discontinuation prior to a non-emergent cardiac or non-cardiac surgery is 3, 5 and 7 days for ticagrelor, clopidogrel and prasugrel, respectively [1, 2].

Ticagrelor exerts more potent antiplatelet effect compared with clopidogrel, while its antiaggregatory action is comparable to one observed with prasugrel [15, 16, 17]. Clinical superiority of ticagrelor over clopidogrel has been proven in the Platelet Inhibition and Patient Outcomes (PLATO) study [5]. This was a double-blind, randomized trial, which included 18,624 patients with ACS. Patients receiving ticagrelor had a significant reduction in occurrence of composite of death from vascular causes, myocardial infarction (MI), or stroke, compared with patients on clopidogrel (9.8% vs. 11.7%, *p *< 0.001). Additionally, ticagrelor-treated patients had lower rates of MI alone (5.8% vs. 6.9%, *p* = 0.005), death from vascular causes (4.0% vs. 5.1%, *p* = 0.001), death from any cause (4.5% vs. 5.9%, *p *< 0.001), and stent thrombosis (1.3% vs. 1.9%, *p* = 0.009). These results were attributed not only to more favorable pharmacokinetics and pharmacodynamics of ticagrelor, but also partially to its pleiotropic effects, including increased plasma concentrations of adenosine [18]. Importantly, ticagrelor exerts consistent antiaggregatory and clinical effects in high-risk groups, i.e., patients with diabetes or chronic kidney disease [19, 20, 21, 22, 23].

### 2.1 Clinical Indications for Ticagrelor

Ticagrelor can be used in numerous clinical scenarios. In the majority of ACS patients ticagrelor is preferred over clopidogrel. Treatment with ticagrelor ought to start with 180 mg loading dose and should be followed by 90 mg twice daily, usually for 12 months [2, 3]. Ticagrelor has class I recommendation in both ST-elevation myocardial infarction (STEMI) and non-ST-elevation ACS (NSTE-ACS). In contrast to prasugrel, it can be administered in patients treated conservatively. In patients with MI at high ischemic risk who have tolerated DAPT without bleeding for 12 months, ticagrelor in reduced maintenance dose of 60 mg twice daily may be preferred over thienopyridines [2]. Notably, according to the Swedish Web-system for Enhancement and Development of Evidence-based Care in Heart Disease Evaluated According to Recommended Therapies (SWEDEHEART) registry, ticagrelor is used in patients with MI over 17 times more often than prasugrel [24].

Clopidogrel remains the P2Y12 receptor inhibitor of choice in patients with chronic coronary syndrome undergoing elective stenting. However, ticagrelor may be considered in specific high-risk situations, such as procedural characteristics associated with high risk of stent thrombosis, complex left main stem, or multivessel stenting, or if DAPT cannot be used because of aspirin intolerance [25]. In elective stenting patients with moderate to high risk of stent thrombosis and indication for chronic anticoagulation, ticagrelor with oral anticoagulant may be considered as a substitute for triple therapy with anticoagulant, aspirin, and clopidogrel [25].

## 3. Bleeding Events in Patients Receiving Ticagrelor

In the PLATO trial use of ticagrelor was associated with significantly higher rate of major and minor bleeding events compared with clopidogrel (16.1% vs. 14.6%, *p* = 0.008) [5]. Patients receiving ticagrelor more frequently suffered from major bleeding not related to coronary-artery bypass grafting (CABG) (4.5% vs. 3.8%, *p* = 0.03), nonintracranial fatal bleeding (0.1% vs. 0.3%, *p* = 0.03) and fatal intracranial bleeding (0.1% vs. 0.01%, *p* = 0.02). Additionally, 8.9% of patients receiving ticagrelor-based DAPT experienced bleeding requiring red-cell transfusion, 5.8% suffered a life-threatening or fatal bleeding, and 7.4% had CABG-related major bleeding [5].

Ticagrelor-related bleeding appears to be dose-dependent and leads to premature interruption of treatment even in up to 7.8% of patients receiving standard maintenance dose (90 mg twice daily). Although bleeding seems to occur less often during treatment with reduced maintenance dose of 60 mg twice daily, it still leads to discontinuation of treatment in 6.2% of patients [26]. This emphasizes a great need for clinical introduction of methods allowing to promptly overcome ticagrelor’s antiplatelet action in case bleeding control or reduction of hemorrhagic risk before urgent surgery is needed.

New ticagrelor-based de-escalation strategies have been proposed recently to reduce the incidence of bleeding in patients receiving ticagrelor. The Ticagrelor with Aspirin or Alone in High-risk Patients after Coronary Intervention (TWILIGHT) trial showed that monotherapy with ticagrelor following 3 months of DAPT after PCI reduces rates of clinically relevant bleeding without increasing the risk of ischemic events [27]. This was also true for patients with NSTE-ACS [28]. Still, as many as 4% of patients from the monotherapy arm experienced the Bleeding Academic Research Consortium (BARC) type 2, 3 or 5 bleeding, compared with 7.1% in the DAPT arm [21]. The pre-specified analysis revealed that patients at high bleeding risk had larger absolute risk reduction in major bleeding than non-high bleeding risk patients [29]. Significant reduction of bleeding risk is also expected from a novel approach currently under investigation in the Evaluation of Safety and Efficacy of Two Ticagrelor-based De-escalation Antiplatelet Strategies in Acute Coronary Syndrome (ELECTRA-SIRIO 2) study. This trial evaluates the impact of monotherapy with reduced maintenance dose of ticagrelor (60 mg twice daily) on bleeding and ischemic events in ACS patients [30, 31].

## 4. Methods of Platelet Function Restoration in Patients Treated with Ticagrelor

### 4.1 Platelet Transfusion

Restoration of thrombocyte function by external supplementation of platelets initially appeared to be both uncomplicated and economical method of overcoming the therapeutical effect of antiplatelet agents [32].

In theory, to achieve maximal reversal of platelet function and to prevent inhibition of transfused thrombocytes, platelet transfusion should occur after the active compounds have been eliminated from circulation [33, 34]. This has been confirmed for prasugrel, in case of which supplemented platelets were partially inhibited by its active metabolite up to 2 hours after prasugrel loading dose, while no significant inhibition was observed starting from 6 hours after a loading dose [35]. Time-dependent effect of platelet transfusion may be even more expressed in subjects receiving ticagrelor. A study by Hansson *et al*. [36] shows that influence of *ex vivo* platelet supplementation on platelet aggregability in blood samples from patients receiving ticagrelor 2 hours prior is limited and lower than in those treated with clopidogrel. Scharbert *et al*. [37] have examined the impact of plasma obtained from P2Y12 receptor antagonist-treated patients on platelet function of subjects not receiving antiplatelet agents. The plasma was collected 3 hours after administration of thienopyridines or ticagrelor. Clopidogrel had no and prasugrel had only mild effect on platelet function of healthy volunteers, as their active metabolites were mostly bound or vanished by the time of assessment. Ticagrelor completely abrogated ADP-mediated platelet activation, and even at low concentrations, it has substantially inhibited platelet aggregation [37]. Platelet rich plasma obtained at 4 hours after the last ticagrelor’s dose also reduces ADP responsiveness of platelets in ticagrelor-naïve patients. This supports the concept that plasma- or platelet-bound ticagrelor and its active metabolite can decrease platelet reactivity of supplemented thrombocytes. Of note, this was not observed with clopidogrel or prasugrel [38]. The Antagonize P2Y12 Treatment Inhibitors by Transfusion of Platelets in an Urgent or Delayed Timing After Acute Coronary Syndrome or Percutaneous Coronary Intervention Presentation-Acute Coronary Syndrome (APTITUDE-ACS) study evaluated influence of *ex vivo* autologous platelet transfusion 4 hours after P2Y12 receptor antagonist loading dose in patients with ACS or undergoing coronary stenting on the restoration of platelet reactivity [39]. In patients receiving clopidogrel transfusion led to a significant 34% relative increase of platelet reactivity according to the vasodilator-stimulated phosphoprotein phosphorylation (VASP) assay (*p* = 0.0008) compared to baseline. In pooled population of patients treated with prasugrel or ticagrelor a 24% relative increase was not statistically significant (*p* = 0.22) [39].

A trial by Kruger *et al*. [40] showed that supplementation of an equivalent of six apheresis platelet units produces a 50% relative reversal of ticagrelor-induced platelet inhibition at 10 hours after the last maintenance dose. The same amount of transfused platelets could lead to reversal exceeding 90% at 24 hours after the last ticagrelor dose [40]. Similar study by Zahar *et al*. [41] confirmed that restoration of platelet function in ticagrelor-treated patients is time-dependent regarding the last dose intake. *In vitro* addition of concentrated platelets at 4 or 6 hours after administration of ticagrelor loading or maintenance dose produced at most 35% of baseline aggregation. Depending on the amount of supplemented platelets, transfusion at 24 hours post-ticagrelor dose generated 59–79% of baseline reactivity, which increased to >85% at 48 hours [41]. Although it is hard to correspond numerical values of platelet reactivity to clinical outcomes, the authors concluded that most likely at least 24 hours from the last ticagrelor dosing might be necessary to observe a clinical improvement related to restoration of platelet function [41]. Contrarily, Teng *et al*. [42] reported that transfusion of autologous platelet apheresis unit (approximately six pooled donor platelet units) failed to restore platelet function even when it was supplemented 48 hours after the last ticagrelor dose.

These pharmacodynamic data have not been verified in an appropriately sized clinical trial so far. Single case reports indicated clinical inefficacy of platelet transfusion for management of major bleeding in ticagrelor-treated patients [43, 44]. Although it appears that platelet supplementation is not very likely to be useful in urgent scenarios (bleeding, need for immediate surgery), it should be considered that currently available studies evaluated surrogate endpoints only.

### 4.2 Other Non-Specific Methods of Platelet Function Restoration

Schoener *et al. *reported potential usefulness of human albumin supplementation in reversal of ticagrelor antiplatelet action*.* In their *in vitro* study, they have attempted to overcome antiaggregatory effect of ticagrelor in patients with ACS using several different strategies [45]. Supplementation of pooled platelets has not improved platelet function according to the VASP assay, while addition of platelet rich plasma managed to significantly increase platelet aggregation (14.8% → 36.7%, *p *< 0.001). Likewise, supplementation of both human serum and human serum albumin significantly limited antiplatelet effect of ticagrelor (11.7% → 54.1%, *p *< 0.001; 8.9% → 48.1 %, *p *< 0.001, respectively). As 99.8% of ticagrelor is bound to plasma proteins [46], the authors hypothesized that due to reversibility of ticagrelor interaction with platelet P2Y12 receptors, with addition of serum proteins (mainly albumin) ticagrelor diffuses from the target receptor. Only the highest tested concentration of human serum albumin (80 g/L) produced statistically significant decrease of platelet inhibition. Trend for restoration was also present for lower concentrations (8–16 g/L) corresponding to doses that are more realistic in a routine practice [45]. This approach has not been tested in a properly sized randomized clinical trial.

An alternative strategy to restore platelet function in patients treated with ticagrelor is to remove the drug from circulation using sorbent hemadsorption [47]. In their study, Angheloiu *et al*. [47] used CytoSorb, a styrene copolymer with bead diameters of 425 to 1000 mm and surface area 850 $m^{2}$/g. It was very efficient in removal of ticagrelor from human plasma, as well whole blood, with almost complete removal of the antiplatelet agent. With the proposed method 99.99% of ticagrelor was removed from freshly separated plasma within 10 hours. Although almost complete removal of ticagrelor required 10 hours, 90% of the drug was removed already at 3 hours, and 98.5% at 7 hours. These results were in line with removal rates obtained in analogous experiment with whole blood, where 94%, 97.5%, and 99% of ticagrelor was removed after 3–4, 7 and 10 hours, respectively [47]. It seems clinically important that the vast majority of ticagrelor elimination occurs during the first hours of hemadsorption, making this strategy potentially useful in acute clinical conditions requiring quick abrogation of ticagrelor-induced platelet inhibition. Of note, this trial has not evaluated the impact of this approach on the latter and this matter warrants elucidation.

Several substances with mechanism of action different from enhancement of ADP-dependent platelet aggregation have been proposed for improvement of hemostasis in patients receiving ticagrelor [48, 49, 50, 51]. In a study with healthy volunteers desmopressin shortened ticagrelor-induced bleeding time, but it was not statistically significant and was not considered clinically relevant. As expected, inhibition of platelet aggregation by ticagrelor was not affected by co-administration of desmopressin [48]. Activated recombinant human factor VII, as well as recombinant human prothrombin were shown to reduce blood loss and bleeding time in ticagrelor-pretreated mice [49]. Recombinant activated factor VII, fibrinogen concentrate and factor XIII concentrate were shown to partly compensate ticagrelor-induced bleeding by acting on fibrin formation and fibrinolysis in an *in vitro* study. Simultaneously, they had no impact on ticagrelor-induced platelet inhibition [50]. There is a case report available on recombinant activated factor VII administration in a patient treated with ticagrelor who required urgent neurosurgery for an intracranial hematoma. Platelet inhibition remained unchanged, but thromboelastometric clotting time was reduced and patient had improved hemostasis. No bleeding complications of surgery occurred, but the patient developed pulmonary embolism secondary to recombinant activated factor VII administration [51].

### 4.3 Bentracimab

**Fig. 2.**
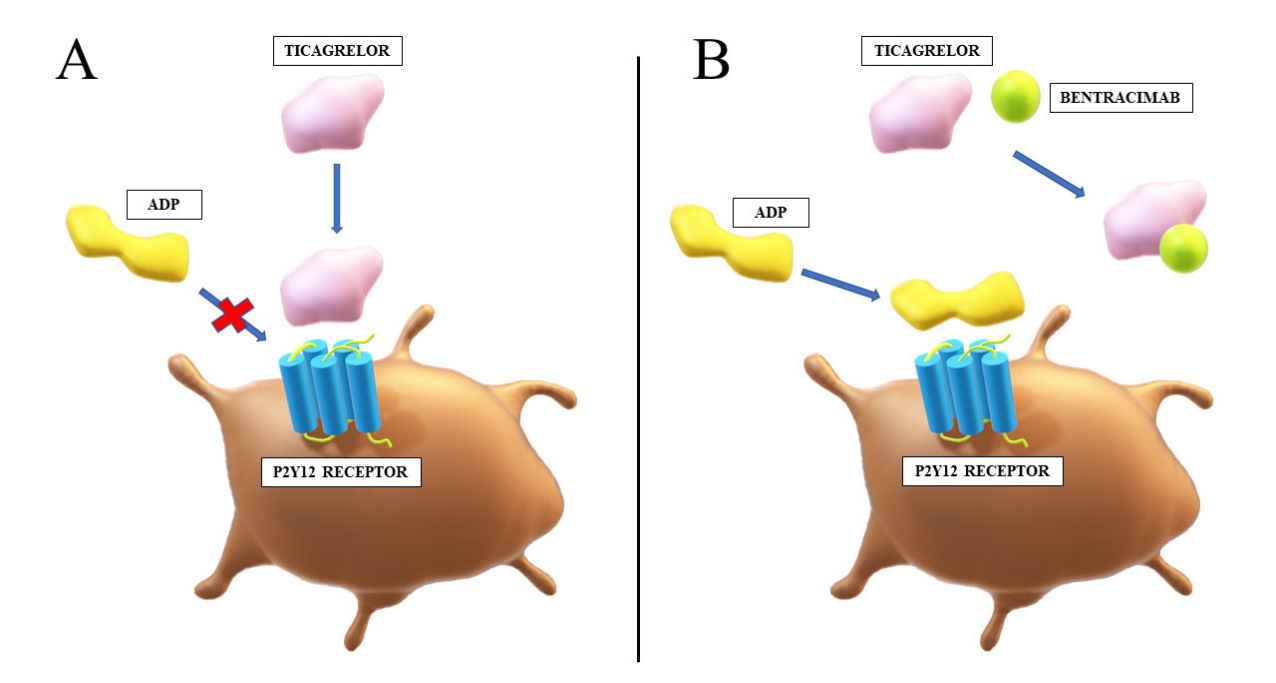
**Mechanism of action of bentracimab**. (A) Ticagrelor reversibly binds to the P2Y12 receptor and inhibits ADP-mediated activation of thrombocytes. (B) Bentracimab binds to ticagrelor with high affinity and specificity. This prevents inhibition of ADP signaling through the P2Y12 receptor by ticagrelor, which restores platelet activity. ADP, adenosine diphosphate.

Bentracimab, formerly known as MEDI2452 or PB2452, is the first specific antidote for ticagrelor, and in fact against any antiplatelet agent. It is an antigen-binding fragment (Fab) that displays 100-fold greater affinity for ticagrelor and its active metabolite (AR-C124910XX) than for their target, platelet P2Y12 receptor [46]. The antidote is highly specific and does not bind to substances of structure similar to ticagrelor or AR-C124910XX, such as adenosine, ADP or adenosine triphosphate. Bentracimab binds to both ticagrelor and its active metabolite in 1:1 ratio, and reverses their antiplatelet effect in a concentration-dependent manner (Fig. 2). Bentracimab also reduces concentration of free ticagrelor, which inversely correlates with recovery of ADP-induced platelet aggregation [52]. This antidote is not expected to be effective against clopidogrel or prasugrel, which are irreversible P2Y12 receptor inhibitors.

In murine model bentracimab enables a 94% *in vivo* reversal of ticagrelor-induced platelet inhibition, with a full onset of reversal occurring 30 minutes after administration of the antidote. This leads to reduction of blood loss and shortens bleeding time to levels similar to ticagrelor-naïve mice [46]. In a porcine model bentracimab dosing leads to complete clearance of free ticagrelor and AR-C124910XX within 5 minutes of administration [53]. This produces platelet function recovery, however with some delay, as 60 minutes are needed to restore ADP-mediated platelet aggregation in ticagrelor-treated pigs. These pharmacokinetic and pharmacodynamic effects translate into numerical increase in survival and reduction of blood loss in pigs with induced major bleed (excision of a liver lobe), however, without a statistical significance. Still, bentracimab substantially reduced the rate of mean arterial pressure decrease over time in these animals [53].

Safety, efficacy, and pharmacokinetic profile of bentracimab in healthy volunteers pretreated with ticagrelor for 48 hours, were evaluated in a single-center, randomized, double-blind, placebo-controlled, phase 1 trial performed by Bhatt *et al*. [54]. A total of 64 volunteers were randomized to placebo (n = 16, mean age: 34.0 ± 8.3 years) or ticagrelor (n = 48, mean age: 30.5 ± 8.8 years). In this first in-human study of bentracimab no dose-limiting toxic effects, infusion-related reactions, deaths, adverse events leading to discontinuation of the trial drug or hospitalization, were observed. There were also no severe adverse events associated with bentracimab administration. Volunteers receiving bentracimab had more adverse events than those receiving placebo (35% vs. 12%). They were predominantly clinically insignificant, and included: bruising at infusion site (8%), reaction at medical device site (6%), bruising at vessel puncture site (4%), extravasation at infusion site (4%), abdominal pain (2%), acute respiratory failure (2%), alcohol poisoning (2%), aspiration pneumonia (2%), blood in urine (2%), conjunctivitis (2%), dizziness (2%), gastroenteritis (2%), hematuria (2%), nasopharyngitis (2%), oropharyngeal pain (2%), reaction at infusion site (2%), skin abrasion (2%), streptococcal pharyngitis (2%), and upper limb fracture (2%). Additionally, 12% of volunteers became positive for antidrug antibodies after they were administered with bentracimab, while 31% of participants receiving the antidote had preexisting antibodies against bentracimab. All antibody titers were low and had no observed effect on safety or efficacy of bentracimab [54].

Abovementioned study evaluated several dosing regimens of bentracimab. The greater were the bolus and duration of infusion, the more rapid and sustained ticagrelor reversal was. With maximal bolus of 6 g followed by a 12-hour or 16-hour infusion up to total dose of 18 g, bentracimab provided reversal of ticagrelor-induced platelet inhibition within 5 minutes after initiation of the infusion, that was maintained for 16 to 24 hours [54]. These pharmacodynamic effects in healthy volunteers were documented with three different platelet function tests. Reversal of platelet aggregation was ≥80% of baseline, >180 units, and nearly 100% of baseline, for light transmission aggregometry, the VerifyNow assay, and the VASP assay, respectively. High correlation was observed for results obtained with different platelet function tests, with r values exceeding 0.9 (*p *< 0.001 for all comparisons). There was no rebound in platelet aggregation after cessation of bentracimab infusion [54].

Consistent results were recently made available from a phase 2b trial assessing safety and efficacy of bentracimab in reversing the antiplatelet effect of ticagrelor [55]. Peer-reviewed results of this trial are not available yet, however, main findings have been made public. In this study 205 healthy volunteers pretreated with ticagrelor for 48 hours were randomized in a 3:1 ratio to either bentracimab (n = 154) or placebo (n = 51). Study participants were older than in the previous phase 1 trial (mean age: 61 years), half of them were female, and 59% had mild and 9% moderate chronic kidney disease. Volunteers allocated to bentracimab had significantly lower platelet inhibition according to the VerifyNow assay within the first 4 hours of the antidote infusion compared with volunteers receiving placebo (*p *< 0.0001). The increase of platelet activation occurred as early as 5–10 minutes post-infusion, and no noticeable rebound in regard to platelet activation was present after cessation of the infusion. No thrombotic events or deaths occurred during 48 hours of follow up [55].

Clinical evaluation of bentracimab is currently under investigation in the Bentracimab in Ticagrelor-treated Patients With Uncontrolled Major or Life-Threatening Bleeding or Requiring Urgent Surgery or Invasive Procedure (REVERSE-IT) study. It is a phase 3, open-label, single arm trial including on-ticagrelor patients with uncontrolled major or life-threatening bleeding, or requiring urgent surgery or invasive procedure [56]. The trial is expected to include target 200 participants by the end of year 2023.

It is not possible to precisely estimate the number of patients who may benefit from bentracimab. It is highly probable that its common use may be restricted due to limited availability and resources. This issue has been observed with idarucizumab and andexanet alfa, specific antidotes for dabigatran, and rivaroxaban and apixaban, respectively. According to the Premier Healthcare Database accounting approximately 25% of all inpatient admissions in the United States, among 550,663 patients hospitalized due to life-threatening bleeding between May 2018 and June 2019, only 407 received idarucizumab and 151 were administered with andexanet alfa [57].

## 5. Conclusions

Currently there are no commercially available methods to reverse ticagrelor-induced platelet inhibition when timely reversal is required in case of a severe hemorrhage or a need for an urgent surgery (Table 1). Non-specific methods aiming to overcome antiplatelet effect of ticagrelor are ineffective or have not been verified in an adequate clinical trial. Bentracimab, the first specific antidote against ticagrelor, provides rapid and sustained restoration of platelet aggregation, but its clinical efficacy and safety are yet to be confirmed in a phase 3 trial, that is currently ongoing.

**Table 1.** **Proposed methods for platelet reactivity restoration in ticagrelor-treated patients**.

Strategy	Specificity for ticagrelor	Key points
Platelet transfusion	No	∙ magnitude of platelet reactivity restoration depends on time from the last ticagrelor dose and amount of supplemented platelets
		∙ at least 24–48 hours from the last ticagrelor dose are necessary to obtain pharmacodynamic effect of platelet transfusion
		∙ efficacy of this strategy has not been verified in a randomized study with clinical endpoints
Human albumin supplementation	No	∙ supplementation of human serum albumin enables restoration of platelet reactivity to approximately half of the baseline values evaluated before ticagrelor administration
		∙ concentrations of human serum albumin that are realistic in a routine practice showed only a trend for restoration of platelet function, without a statistical significance
		∙ efficacy of this strategy has not been verified in a randomized study with clinical endpoints
Hemadsorption	No	∙ ticagrelor is removed from the plasma/whole blood with use of sorbent hemadsorption
		∙ almost complete removal of ticagrelor lasts for 10 hours, however approximately 90% of the drug is removed within the first 3 hours
		∙ pharmacodynamic effects of this strategy warrant further research
		∙ efficacy of this strategy has not been verified in a randomized study with clinical endpoints
Bentracimab	Yes	∙ the first specific antidote for an antiplatelet agent
		∙ an antigen-binding fragment that is highly specific for ticagrelor and its active metabolite
		∙ quick onset of action and effective restoration of platelet reactivity
		∙ currently under investigation in a phase 3 trial

With the shortest time between the last dose and offset of antiplatelet effect, a specific antidote being under advanced development, and very strong recommendations from the cardiac societies, ticagrelor should be considered as P2Y12 receptor inhibitor of choice in the majority of ACS patients. Nevertheless, novel strategies to reduce bleeding burden in patients on ticagrelor are urgently needed, as clinical verification of its first antidote.

## References

[1] Valgimigli M, Bueno H, Byrne RA, Collet JP, Costa F, Jeppsson A (2018). 2017 ESC focused update on dual antiplatelet therapy in coronary artery disease developed in collaboration with EACTS: The Task Force for dual antiplatelet therapy in coronary artery disease of the European Society of Cardiology (ESC) and of the European Association for Cardio-Thoracic Surgery (EACTS). *European Heart Journal*.

[2] Neumann FJ, Sousa-Uva M, Ahlsson A, Alfonso F, Banning AP, Benedetto U (2019). 2018 ESC/EACTS Guidelines on myocardial revascularization. *European Heart Journal*.

[3] Collet JP, Thiele H, Barbato E, Barthélémy O, Bauersachs J, Bhatt DL (2021). 2020 ESC Guidelines for the management of acute coronary syndromes in patients presenting without persistent ST-segment elevation. *European Heart Journal*.

[4] Kubica J, Adamski P, Ładny JR, Kaźmierczak J, Fabiszak T, Filipiak KJ (2022). Pre-hospital treatment of patients with acute coronary syndrome: Recommendations for medical emergency teams. Expert position update 2022. *Cardiology Journal*.

[5] Wallentin L, Becker RC, Budaj A, Cannon CP, Emanuelsson H, Held C (2009). Ticagrelor versus Clopidogrel in Patients with Acute Coronary Syndromes. *New England Journal of Medicine*.

[6] Wiviott SD, Braunwald E, McCabe CH, Montalescot G, Ruzyllo W, Gottlieb S (2007). Prasugrel versus Clopidogrel in Patients with Acute Coronary Syndromes. *New England Journal of Medicine*.

[7] Turgeon RD, Koshman SL, Dong Y, Graham MM (2022). P2Y12 inhibitor adherence trajectories in patients with acute coronary syndrome undergoing percutaneous coronary intervention: prognostic implications. *European Heart Journal*.

[8] Adamski P, Ostrowska M, Navarese EP, Kubica J (2021). Pharmacodynamic and clinical efficacy of reduced ticagrelor maintenance doses in patients with coronary artery disease. *Current Medical Research and Opinion*.

[9] Coons JC, Schwier N, Harris J, Seybert AL (2014). Pharmacokinetic evaluation of prasugrel for the treatment of myocardial infarction. *Expert Opinion on Drug Metabolism and Toxicology*.

[10] Teng R (2015). Ticagrelor: Pharmacokinetic, Pharmacodynamic and Pharmacogenetic Profile: an Update. *Clinical Pharmacokinetics*.

[11] Kristensen SD, Knuuti J, Saraste A, Anker S, Bøtker HE, Hert SD (2014). 2014 ESC/ESA Guidelines on non-cardiac surgery: cardiovascular assessment and management: The Joint Task Force on non-cardiac surgery: cardiovascular assessment and management of the European Society of Cardiology (ESC) and the European Society of Anaesthesiology (ESA). *European Heart Journal*.

[12] Kubica J, Adamski P, Ostrowska M, Sikora J, Kubica JM, Sroka WD (2016). Morphine delays and attenuates ticagrelor exposure and action in patients with myocardial infarction: the randomized, double-blind, placebo-controlled IMPRESSION trial. *European Heart Journal*.

[13] Adamski P, Sikora J, Laskowska E, Buszko K, Ostrowska M, Umińska JM (2017). Comparison of bioavailability and antiplatelet action of ticagrelor in patients with ST-elevation myocardial infarction and non-ST-elevation myocardial infarction: a prospective, observational, single-centre study. *PLoS ONE*.

[14] Adamski P, Buszko K, Sikora J, Niezgoda P, Fabiszak T, Ostrowska M (2019). Determinants of high platelet reactivity in patients with acute coronary syndromes treated with ticagrelor. *Scientific Reports*.

[15] Storey RF, Angiolillo DJ, Patil SB, Desai B, Ecob R, Husted S (2010). Inhibitory Effects of Ticagrelor Compared with Clopidogrel on Platelet Function in Patients with Acute Coronary Syndromes: the PLATO (PLATelet inhibition and patient Outcomes) PLATELET substudy. *Journal of the American College of Cardiology*.

[16] Parodi G, Valenti R, Bellandi B, Migliorini A, Marcucci R, Comito V (2013). Comparison of Prasugrel and Ticagrelor Loading Doses in ST-Segment Elevation Myocardial Infarction Patients: RAPID (Rapid Activity of Platelet Inhibitor Drugs) primary PCI study. *Journal of the American College of Cardiology*.

[17] Adamski P, Barańska M, Ostrowska M, Kuliczkowski W, Buszko K, Kościelska-Kasprzak K (2022). Diurnal Variability of Platelet Aggregation in Patients with Myocardial Infarction Treated with Prasugrel and Ticagrelor. *Journal of Clinical Medicine*.

[18] Adamski P, Koziński M, Ostrowska M, Fabiszak T, Navarese EP, Paciorek P (2014). Overview of pleiotropic effects of platelet P2Y12 receptor inhibitors. *Thrombosis and Haemostasis*.

[19] Singam NSV, AlAdili B, Amraotkar AR, Coulter AR, Singh A, Kulkarni S (2022). In-vivo platelet activation and aggregation during and after acute atherothrombotic myocardial infarction in patients with and without Type-2 diabetes mellitus treated with ticagrelor. *Vascular Pharmacology*.

[20] Wang H, Qi J, Li Y, Tang Y, Li C, Li J (2018). Pharmacodynamics and pharmacokinetics of ticagrelor vs. clopidogrel in patients with acute coronary syndromes and chronic kidney disease. *British Journal of Clinical Pharmacology*.

[21] James S, Angiolillo DJ, Cornel JH, Erlinge D, Husted S, Kontny F (2010). Ticagrelor vs. clopidogrel in patients with acute coronary syndromes and diabetes: a substudy from the PLATelet inhibition and patient Outcomes (PLATO) trial. *European Heart Journal*.

[22] James S, Budaj A, Aylward P, Buck KK, Cannon CP, Cornel JH (2010). Ticagrelor Versus Clopidogrel in Acute Coronary Syndromes in Relation to Renal Function. *Circulation*.

[23] Franchi F, James SK, Ghukasyan Lakic T, Budaj AJ, Cornel JH, Katus HA (2019). Impact of Diabetes Mellitus and Chronic Kidney Disease on Cardiovascular Outcomes and Platelet P2Y_12 Receptor Antagonist Effects in Patients With Acute Coronary Syndromes: Insights From the PLATO Trial. *Journal of the American Heart Association*.

[24] Venetsanos D, Träff E, Erlinge D, Hagström E, Nilsson J, Desta L (2021). Prasugrel versus ticagrelor in patients with myocardial infarction undergoing percutaneous coronary intervention. *Heart*.

[25] Knuuti J, Wijns W, Saraste A, Capodanno D, Barbato E, Funck-Brentano C (2020). 2019 ESC Guidelines for the diagnosis and management of chronic coronary syndromes. *European Heart Journal*.

[26] Bonaca MP, Bhatt DL, Cohen M, Steg PG, Storey RF, Jensen EC (2015). Long-Term Use of Ticagrelor in Patients with Prior Myocardial Infarction. *New England Journal of Medicine*.

[27] Mehran R, Baber U, Sharma SK, Cohen DJ, Angiolillo DJ, Briguori C (2019). Ticagrelor with or without Aspirin in High-Risk Patients after PCI. *New England Journal of Medicine*.

[28] Baber U, Dangas G, Angiolillo DJ, Cohen DJ, Sharma SK, Nicolas J (2020). Ticagrelor alone vs. ticagrelor plus aspirin following percutaneous coronary intervention in patients with non-ST-segment elevation acute coronary syndromes: TWILIGHT-ACS. *European Heart Journal*.

[29] Escaned J, Cao D, Baber U, Nicolas J, Sartori S, Zhang Z (2021). Ticagrelor monotherapy in patients at high bleeding risk undergoing percutaneous coronary intervention: TWILIGHT-HBR. *European Heart Journal*.

[30] Kubica J, Adamski P, Gorog DA, Kubica A, Jilma B, Budaj A (2021). Low-dose ticagrelor with or without acetylsalicylic acid in patients with acute coronary syndrome: Rationale and design of the ELECTRA-SIRIO 2 trial. *Cardiology Journal*.

[31] Kubica J, Adamski P, Niezgoda P, Kubica A, Podhajski P, Barańska M (2021). A new approach to ticagrelor-based de-escalation of antiplatelet therapy after acute coronary syndrome. A rationale for a randomized, double-blind, placebo-controlled, investigator-initiated, multicenter clinical study. *Cardiology Journal*.

[32] Hobl EL, Derhaschnig U, Firbas C, Schoergenhofer C, Schwameis M, Jilma B (2013). Reversal strategy in antagonizing the P2Y12-inhibitor ticagrelor. *European Journal of Clinical Investigation*.

[33] Godier A, Garrigue D, Lasne D, Fontana P, Bonhomme F, Collet J (2019). Management of antiplatelet therapy for non elective invasive procedures of bleeding complications: proposals from the French working group on perioperative haemostasis (GIHP), in collaboration with the French Society of Anaesthesia and Intensive Care Medicine (SFAR). *Anaesthesia Critical Care and Pain Medicine*.

[34] Godier A, Albaladejo P (2020). Management of Bleeding Events Associated with Antiplatelet Therapy: Evidence, Uncertainties and Pitfalls. *Journal of Clinical Medicine*.

[35] Zafar MU, Santos-Gallego C, Vorchheimer DA, Viles-gonzalez JF, Elmariah S, Giannarelli C (2013). Platelet function normalization after a prasugrel loading-dose: time-dependent effect of platelet supplementation. *Journal of Thrombosis and Haemostasis*.

[36] Hansson EC, Shams Hakimi C, Åström-Olsson K, Hesse C, Wallén H, Dellborg M (2014). Effects of ex vivo platelet supplementation on platelet aggregability in blood samples from patients treated with acetylsalicylic acid, clopidogrel, or ticagrelor. *British Journal of Anaesthesia*.

[37] Scharbert G, Wetzel L, Schrottmaier WC, Kral JB, Weber T, Assinger A (2015). Comparison of patient intake of ticagrelor, prasugrel, or clopidogrel on restoring platelet function by donor platelets. *Transfusion*.

[38] Bertling A, Fender AC, Schüngel L, Rumpf M, Mergemeier K, Geißler G (2018). Reversibility of platelet P2Y12 inhibition by platelet supplementation: ex vivo and in vitro comparisons of prasugrel, clopidogrel and ticagrelor. *Journal of Thrombosis and Haemostasis*.

[39] O’Connor SA, Amour J, Mercadier A, Martin R, Kerneis M, Abtan J (2015). Efficacy of ex vivo autologous and in vivo platelet transfusion in the reversal of P2Y12 inhibition by clopidogrel, prasugrel, and ticagrelor: the APTITUDE study. *Circulation: Cardiovascular Interventions*.

[40] Kruger P, Hirsh J, Bhagirath V, Xu K, Dale B, de Vries T (2018). In Vitro Reversal of the Anti-Aggregant Effect of Ticagrelor Using Untreated Platelets. *Thrombosis and Haemostasis*.

[41] Zafar MU, Smith DA, Baber U, Sartori S, Chen K, Lam DW (2017). Impact of Timing on the Functional Recovery Achieved with Platelet Supplementation after Treatment with Ticagrelor. *Circulation: Cardiovascular Interventions*.

[42] Teng R, Carlson GF, Nylander S, Andersson TLG (2016). Effects of autologous platelet transfusion on platelet inhibition in ticagrelor-treated and clopidogrel-treated subjects. *Journal of Thrombosis and Haemostasis*.

[43] Godier A, Taylor G, Gaussem P (2015). Inefficacy of Platelet Transfusion to Reverse Ticagrelor. *New England Journal of Medicine*.

[44] Filaire L, Pham DT, d’Ostrevy N, Tran HT, Camilleri L, Azarnoush K (2017). Inefficacy of Platelet Transfusion in a Heart Transplant Patient under Continuous Ticagrelor. *Journal of Cardiothoracic and Vascular Anesthesia*.

[45] Schoener L, Jellinghaus S, Richter B, Pfluecke C, Ende G, Christoph M (2017). Reversal of the platelet inhibitory effect of the P2Y12 inhibitors clopidogrel, prasugrel, and ticagrelor in vitro: a new approach to an old issue. *Clinical Research in Cardiology*.

[46] Teng R, Oliver S, Hayes MA, Butler K (2010). Absorption, Distribution, Metabolism, and Excretion of Ticagrelor in Healthy Subjects. *Drug Metabolism and Disposition*.

[47] Angheloiu GO, Gugiu GB, Ruse C, Pandey R, Dasari RR, Whatling C (2017). Ticagrelor Removal from Human Blood. *JACC: Basic to Translational Science*.

[48] Teng R, Mitchell PD, Butler K (2014). The effect of desmopressin on bleeding time and platelet aggregation in healthy volunteers administered ticagrelor. *Journal of Clinical Pharmacy and Therapeutics*.

[49] Pehrsson S, Hansson K, Nelander K, Nylander S (2016). Boosting the coagulation restores haemostasis in ticagrelor-treated mice. *Blood Coagulation and Fibrinolysis*.

[50] Calmette L, Martin AC, Le Bonniec B, Zlotnik D, Gouin-Thibault I, Bachelot-Loza C (2017). Ticagrelor reversal: *in vitro* assessment of four haemostatic agents. *Journal of Clinical Pathology*.

[51] Godier A, Dupont M, Desilles J, Le Guerinel C, Taylor G, Perrin M (2018). Successful Use of Recombinant Activated Factor VII to Reverse Ticagrelor-Induced Bleeding Risk: a Case Report. *TH Open*.

[52] Buchanan A, Newton P, Pehrsson S, Inghardt T, Antonsson T, Svensson P (2015). Structural and functional characterization of a specific antidote for ticagrelor. *Blood*.

[53] Pehrsson S, Johansson KJ, Janefeldt A, Sandinge A, Maqbool S, Goodman J (2017). Hemostatic effects of the ticagrelor antidote MEDI2452 in pigs treated with ticagrelor on a background of aspirin. *Journal of Thrombosis and Haemostasis*.

[54] Bhatt DL, Pollack CV, Weitz JI, Jennings LK, Xu S, Arnold SE (2019). Antibody-Based Ticagrelor Reversal Agent in Healthy Volunteers. *The New England Journal of Medicine*.

[55] (2022). Summary of Phase 2B Study to Evaluate the Efficacy of PB2452 in Reversal of Ticagrelor in Subjects Aged 50–80. https://www.acc.org/latest-in-cardiology/clinical-trials/2022/04/01/03/34/bentracimab.

[56] (2020). Characteristics of the REVERSE-IT study. https://clinicaltrials.gov/ct2/show/NCT04286438.

[57] Spyropoulos AC, Hartaigh BÓ, Cao Z, Caberwal H, Lipkin C, Petrini M (2022). Costs and Healthcare Resource Utilization Associated with Idarucizumab or Andexanet alfa Oral Anticoagulant Reversal in Patients Hospitalized with Life-Threatening Bleeds. *Clinical and Applied Thrombosis/Hemostasis*.

